# Molecular-resolution imaging of pentacene on KCl(001)

**DOI:** 10.3762/bjnano.3.20

**Published:** 2012-02-29

**Authors:** Julia L Neff, Jan Götzen, Enhui Li, Michael Marz, Regina Hoffmann-Vogel

**Affiliations:** 1 Physikalisches Institut and DFG-Center for Functional Nanostructures, Karlsruhe Institute of Technology, Wolfgang-Gaede-Str. 1, 76128 Karlsruhe, Germany; 2 Mechanical Engineering and Materials Science, Yale University, CT 06511, USA; 3 National Institute for Materials Science (NIMS), 1-2-1 Sengen, Tsukuba, Ibaraki, Japan

**Keywords:** KCl, molecular growth, pentacene, scanning force microscopy, self-assembly

## Abstract

The growth of pentacene on KCl(001) at submonolayer coverage was studied by dynamic scanning force microscopy. At coverages below one monolayer pentacene was found to arrange in islands with an upright configuration. The molecular arrangement was resolved in high-resolution images. In these images two different types of patterns were observed, which switch repeatedly. In addition, defects were found, such as a molecular vacancy and domain boundaries.

## Introduction

To understand the functionalization of surfaces with molecular building blocks, an important step is to study the self-assembly of molecules. Scanning tunneling microscopy (STM) enables such studies on conductive surfaces [1–2]. On metallic surfaces, molecular growth is usually governed by strong adsorbate–substrate interactions. However, for some applications in the field of thin-film electronic devices, insulating substrates are required in order to decouple the molecular structure from the substrate. On insulators the interaction of the molecules with the substrate is much weaker than on metals because the partial transfer of electrons is expected to be weak, such that the interaction is dominated by van der Waals and electrostatic interactions, as opposed to chemical bonding. A unique tool to investigate the thin-film structure of molecules adsorbed on insulating materials is the scanning force microscope (SFM). To date only a limited number of molecules have been studied on insulating substrates, see for example [3–11]. Among the frequently studied organic molecules, pentacene has promising perspectives for thin-film electronic devices due to its high carrier mobility [12]. Besides its high carrier mobility, this π-conjugated organic molecule shows shape anisotropy, which leads to a preferential orientation with respect to the substrate in bulk crystals and crystalline thin films. Shape anisotropy also causes a pronounced anisotropy of the electronic transport properties. Therefore, the electronic properties of pentacene are closely related to its structural order, and a precise control of the molecular packing and the crystalline orientation of thin films is of vital interest for the optimization of organic electronic devices [13].

The adsorption of pentacene on various substrates has been investigated with diffraction methods and STM [14–18]. On single crystalline metal surfaces such as, e.g., Cu(110), Au(111) and Ag(111) [19–24], pentacene forms a wetting layer of flat-lying molecules. These order in a commensurable superstructure with respect to the surface pattern. The growth of multilayers depends on the structural details of the substrate. A recent SFM study has shown the morphology of thin pentacene films on Cu(111) with molecular resolution [25]. On graphite, template-induced growth from one monolayer to thick films was studied by STM [18]. The molecular arrangement even on the top of islands with several nanometers in height appears to be commensurate with the graphite surface. On the more inert SiO_2_, templating is not possible, due to the disordered substrate. The molecules crystallize in an upright configuration from the first layer onward [26]. In this configuration, the long edge of the molecule forms an angle of nearly 90° with the surface. In thin films, the molecules crystallize in a thin-film phase [27] that is similar to the bulk phases [28–29] but shows a different tilting angle. On alkali halides, diffraction measurements and ambient SFM measurements of thick pentacene films show similar phases [30–34]. Single flat-lying molecules on ultrathin NaCl films on Cu(111) have been examined by SFM with unprecedented resolution with the aid of a functionalized tip [35].

In this work, we describe the arrangement of pentacene adsorbed on the KCl(001) single-crystal surface. For submonolayer coverage the molecules form islands with upright ordering. In molecularly resolved images of these islands two different molecular patterns are observed. Furthermore, the high-resolution images show domain boundaries and a defect resulting from a molecular vacancy.

## Experimental

Experiments were carried out in an ultrahigh-vacuum (UHV) variable-temperature SFM (Omicron NanoTechnology GmbH, Taunusstein, Germany) with a base pressure below 3 × 10^−10^ mbar. Terraces separated by atomic steps were obtained by cleavage of atomically clean KCl(001) (Kohrt, Altenholz, Germany). The cuboid KCl crystal was mounted such that its edges were aligned with the unturned slow and fast (x,y) scan directions. This alignment of the crystallographic [100] and [010] directions with the (x,y) directions was double-checked by looking at the KCl step edges. After cleavage of the KCl crystal in air, the crystal was immediately introduced into the UHV chamber. Subsequently it was heated to 400 K for about one hour in order to remove contaminations such as water as well as charge buildup produced during the cleavage process. Pentacene molecules (ABCR, Karlsruhe, Germany, purity >98%) were degassed for several hours at temperatures slightly below the evaporation temperature (508 K). Several angstroms of pentacene were deposited from a resistively heated Knudsen cell, while the surface was kept at room temperature. The rate was approximately 1 Å/min and was monitored by a quartz microbalance. Supersharp silicon cantilevers provided by Nanosensors (Neuchatel, Switzerland) were heated in vacuum to about 390 K to remove contaminants. Frequency-modulation dynamic SFM measurements were carried out by using a phase-locked-loop frequency demodulator from Nanonis (SPECS, Zürich, Switzerland). Typical resonance frequencies *f*_0_ and spring constants *k* of the cantilevers were 160 kHz and 45 N/m, respectively. Samples were investigated at room temperature and afterwards at low temperatures. For the data shown here the sample was cooled to below 28 K and investigated under conditions of a nonconstant thermal drift smaller than 0.1 Å/s. The piezo-scanner calibration was double checked by performing high-resolution measurements on the Si(111) surface. To reduce the influence of long-range electrostatic forces, the tip–sample work-function difference was compensated by application of the appropriate bias voltage to the tip.

## Results and Discussion

**Figure 1:**
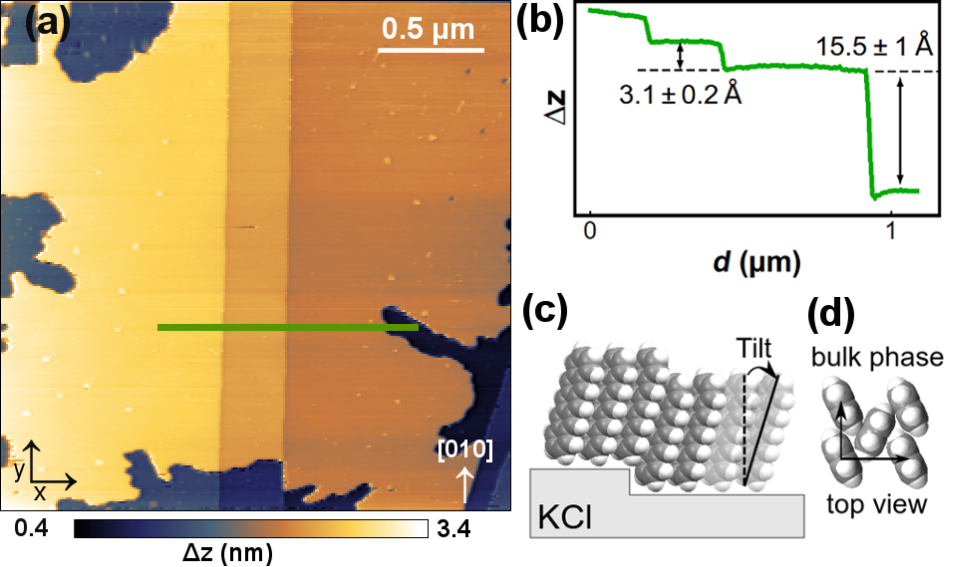
(a) SFM image showing part of a large pentacene island that overgrows two monoatomic substrate steps. *f*_0_ = 160.440 kHz, Δ*f* = −1.541 Hz, oscillation amplitude *A* ≈ 125 nm, *V*_bias_ = −1.875 V. (b) Cross section along the line in (a) cutting two step edges and the island border. (c) Sketch of the upright alignment of the pentacene molecules. (d) Top view of the herringbone arrangement of pentacene in the bulk phase.

Figure 1a shows the KCl(001) surface with submonolayer coverage of pentacene molecules forming an extended island over several microns. The island displays an apparent height of 15.5 ± 1 Å which corresponds to the van der Waals length of the molecule, indicating an upright configuration (Figure 1b). The height of the molecular steps was cross-calibrated by comparison with single KCl steps. Another indication for an upright configuration of the molecules is their smooth growth over substrate step edges. The steps are clearly visible through the molecular film (Figure 1a), which also suggests a high crystallinity of the molecular islands. As mentioned before, pentacene films of several hundred nanometers in thickness order into crystalline layers on alkali halides [30,32–34]. Additionally, pentacene films of approximately 30 nm as well as 100 nm thickness have been found to grow epitaxially on KCl(001) in ambient-pressure SFM and diffraction studies [32–33]. Depending on the substrate temperature during deposition the pentacene films consist of varying fractions of bulk and thin-film phases, in which for higher substrate temperature the bulk-phase fraction dominates [33]. While the bulk phase shows an interlayer distance of 14.1 Å [28–29], the interlayer distance of the thin-film phase on KCl(001) is increased to 15.4 Å [32]. This difference is too small to draw a final conclusion based on SFM measurements, but our results hint at a thin-film-phase configuration. Since already at submonolayer coverage the molecules are arranged in this upright configuration, our results demonstrate that the molecule–substrate interaction is indeed weak compared to the intermolecular interaction. Figure 1c illustrates the upright ordering of the molecules. For comparison we have added a top-view sketch of the well-known bulk phase, showing the herringbone arrangement that the molecules assume to optimize the π-stacking (Figure 1d). The thin-film phase differs in the top-view only slightly from the bulk phase.

**Figure 2:**
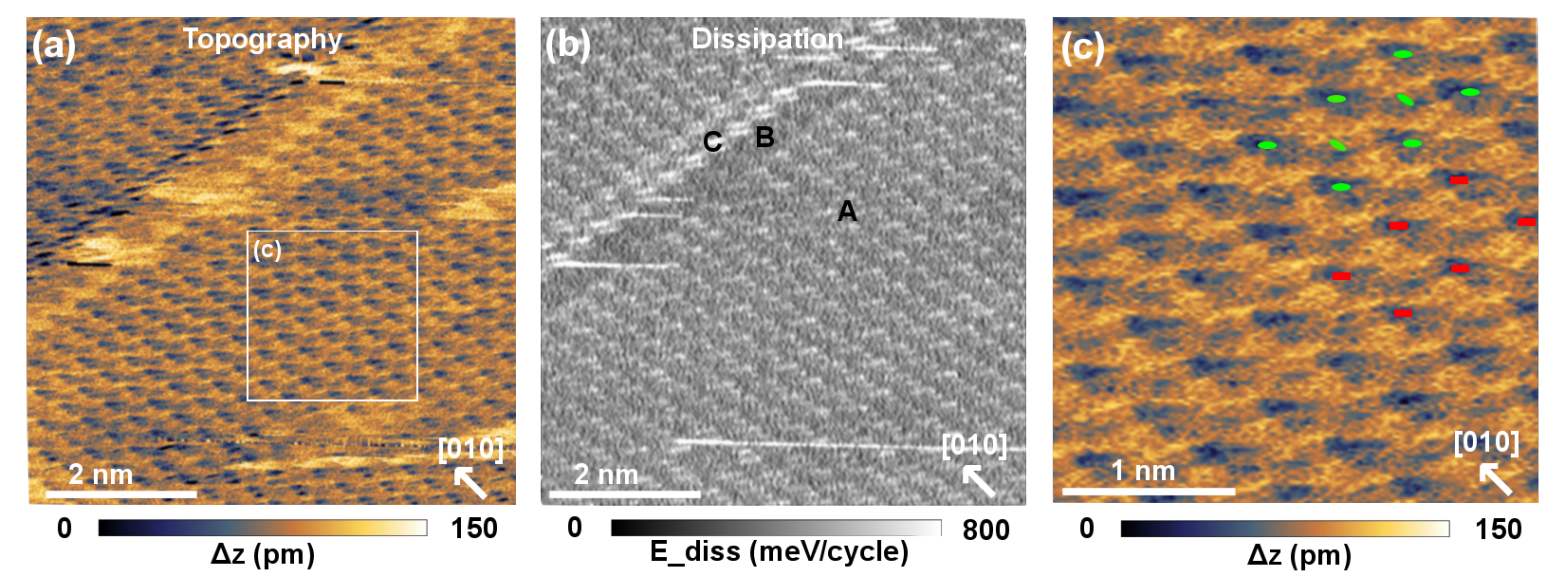
Pattern I. Imaged with an angle of 45°. (a) Topograph. (b) Simultaneously acquired dissipation signal. (c) Magnification of the section in (a) on which two possible molecular arrangements are displayed. Parameters: *f*_0_ = 160.440 kHz, Δ*f* = −1.541 Hz, *A* ≈ 125 nm, *V*_bias_ = −1.895 V.

**Figure 3:**
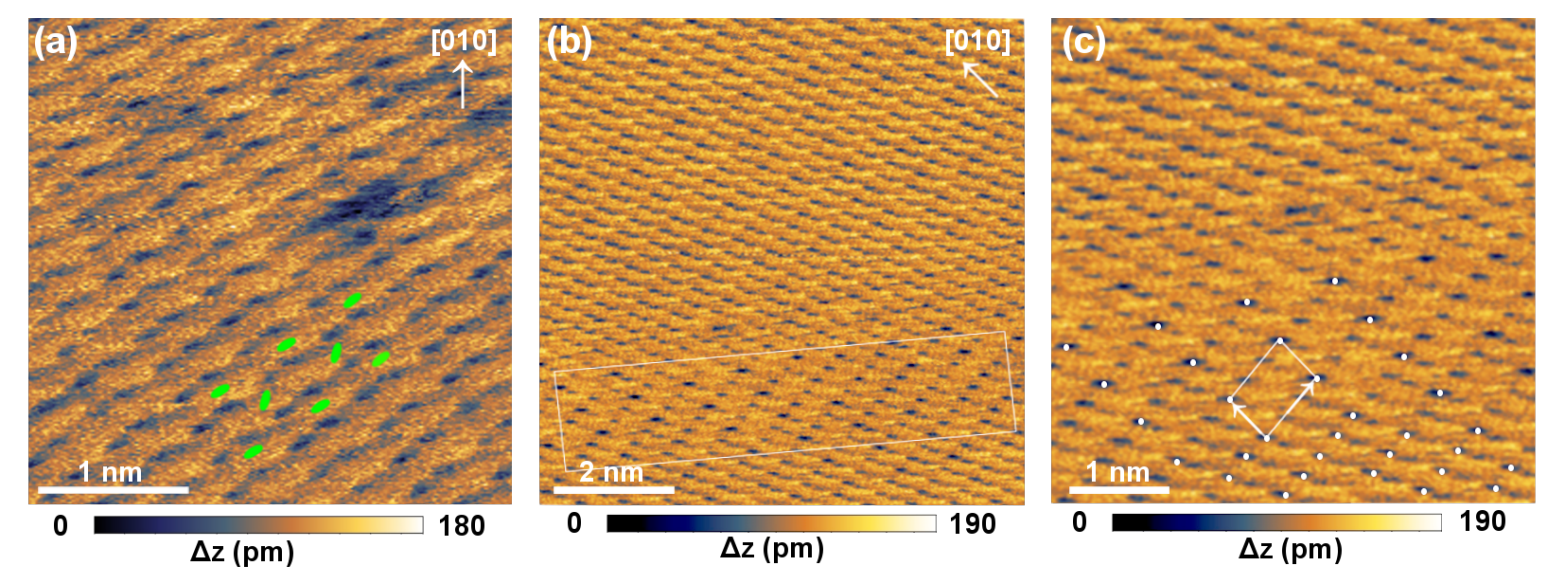
Pattern II. (a) Image displaying a defect. (b) Imaged with an angle of 45°. (c) Magnification of the contrast change in (b) with an illustration of the 3 × 1 superstructure. Parameters: *f*_0_ = 160.440 kHz, Δ*f* = −1.541 Hz, *A* ≈ 125 nm, *V*_bias_ = −1.895 V.

Figure 2 and Figure 3 display molecularly resolved images providing more details about the molecular configuration obtained in this study. Mainly two different types of patterns (I, II) are observed. During imaging at the same frequency shift, the images change repeatedly between these two patterns. Pattern I is characterized by a nearly square surface unit cell (Figure 2c). The molecular unit cell is roughly aligned with the [010] and [100] directions of the KCl substrate. The difference between the experimentally observed alignment and the expected one is consistent with thermal drift. In Figure 2c two possible molecular arrangements are displayed. For both arrangements the molecules have been associated with the dark features of the image, as is typical for inverted contrast, but an association with the bright features is also possible. The red model is obtained when each dark feature is associated with one molecule. The green model is the one expected from X-ray diffraction studies [32–33]. In STM and SFM measurements of upright-standing pentacene molecules (see, e.g., [17]) the contrast of the turned molecule in the center of the unit cell is often weaker. This could also be the case here such that no dark feature is observed in the center of the unit cell. In particular if one or several molecules are located on the tip apex and contribute to the imaging forces, the contrast could strongly depend on the relative orientation of tip and surface molecules.

For pattern II (Figure 3a) rather small features are observed compared to the size of the molecule (van der Waals dimensions for 98% electron density contours: 15.5 Å × 6.3 Å × 2.4 Å [22]). Consequently, if the structure derived from X-ray diffraction measurements is superimposed on the SFM image, the unit cell has a substructure. This could be caused by a multiple tip. For pattern II, we associate the molecules with the bright features of the contrast. The repeated changes between pattern I and II could then be explained by contrast inversion or a tip switch due to pick-up or drop-off of a molecule. In Figure 3b a change of the pattern occurs in the lower part of the scanned area. This change is not caused by a tip change, as the border between the original and the modified pattern does not correspond to a line scan but occurs at an angle with respect to the fast-scan direction. In the modification of pattern II, marked by a rectangle, some of the dark features appear less pronounced while others appear more pronounced. In some parts, every third dark feature appears stronger than the other two, i.e., a 3 × 1 superstructure is formed (Figure 3c).

At least for pattern I an alignment along the substrate directions is observed, hinting at epitaxial growth. This is in agreement with the point-on-line epitaxy suggested for thicker pentacene films in the thin-film and bulk phases [32–33].

Additionally, two kinds of defects were observed. For pattern I, a line defect is observed that also follows the KCl [100] direction (Figure 2a and Figure 2b). On the upper side of the line defect the molecular pattern is displaced along the [100] direction compared to the lower side of the defect. The intrinsic distortions of the image make it difficult to estimate the amount of displacement in this direction. The image does not contradict the possibility that the pattern is displaced by a lattice vector of the substrate unit cell, which is much smaller than the molecular unit cell. In that case the line defect could release strain induced by the epitaxy of the molecules on the surface. Another possibility is that the line defect results from a twinned growth. The line defect also has a profound effect on the energy dissipation (Figure 2b). The dissipated energy per oscillation cycle can be estimated by *E*_diss_ ≈ *E*_0_(*A*_exc_ − *A*_exc,0_)/*A*_exc,0_ with *E*_0_ = π*kA*^2^/*Q* [36]. In this formula, *A*_exc,0_ describes the excitation amplitude of the free cantilever and *A*_exc_ the excitation amplitude in the presence of the sample surface. *A* denotes the oscillation amplitude and *Q* the quality factor of the free cantilever. On the undisturbed part of the surface (marked with ‘A’ in Figure 2b) about 250 meV/cycle are additionally dissipated in each unit cell. At the line defect two areas can be distinguished. In one row of unit cells (area B) only the intrinsic dissipation of the cantilever is observed. Whereas, in another row (C) up to 760 meV are additionally dissipated per oscillation cycle. The increased energy dissipation could be due to extra uncompensated electrostatic charge that induces currents in the tip in each oscillation cycle. In this case, we would expect to see strong effects from this charge in the topographic image,which we do not observe. Another possibility is that in the first part of the defect (B) mobile molecules are clamped due to the locally occurring strain, thus resulting in a row of reduced energy dissipation. This would imply that the defect also contains rows of more loosely bound molecules (C), which cause enhanced energy dissipation compared to the undisturbed island. This line defect shows the true molecular resolution of pattern I.

**Figure 4:**
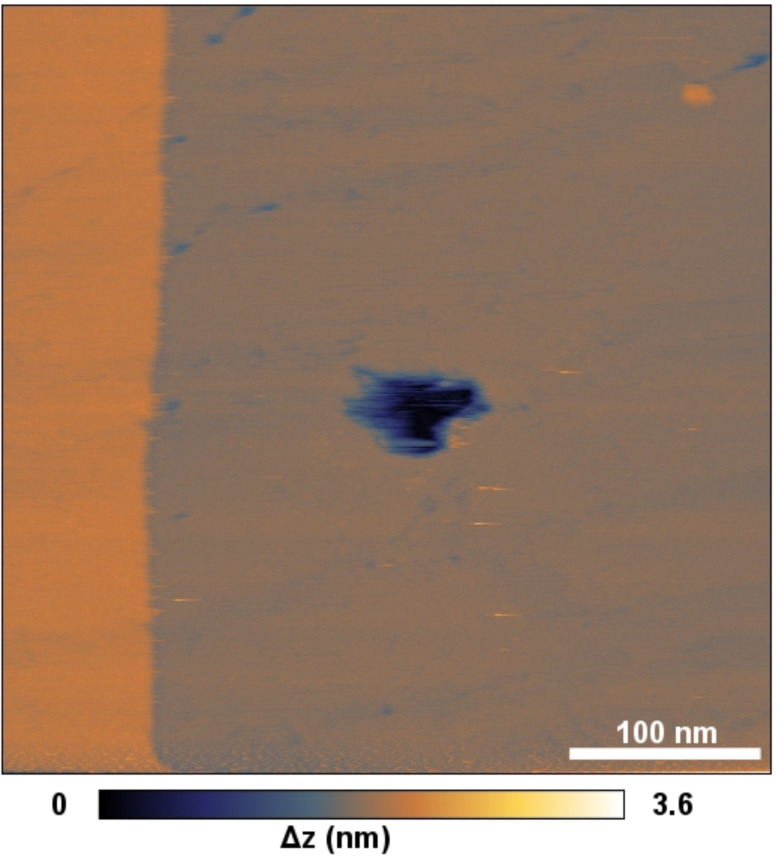
Large-area scan of the area where Figure 2 and Figure 3 were recorded with molecular resolution. *f*_0_ = 160.440 kHz, Δ*f* = −1.541 Hz, *A* ≈ 125 nm, *V*_bias_ = −1.895 V.

For pattern II a point-like defect is displayed in Figure 3a. Here, a darker area is observed with the size of half a unit cell. We attribute this defect to a molecular vacancy caused by one missing molecule. This defect shows that also for pattern II true molecular resolution is obtained. The dissipation contrast in Figure 2b shows that the images were obtained at rather close tip–sample distances. At such small distances the positions of the molecules could be influenced reversibly by the interaction with the SFM tip. However, during the measurements also an irreversible modification of the sample took place. After the data shown in Figure 2 was acquired the molecular resolution was suddenly lost and a hole with a depth of the island was imaged in the area where the previous scans were performed (Figure 4). We exclude the possibility that one of the observed patterns was caused by an irreversible interaction with the scanning tip, because they repeatedly switched.

## Conclusion

The arrangement of pentacene molecules in islands grown on KCl(001) at submonolayer coverage was investigated. It was found that the molecules form islands in an upright configuration. Molecularly resolved images of these islands showed two types of patterns that changed repeatedly. High resolution images revealed further characteristics of the molecular film, such as different defects, e.g., molecular vacancies and domain boundaries.
